# New species of *Plateros* Bourgeois, 1879 (Coleoptera, Lycidae) from Hainan Island, China

**DOI:** 10.3897/zookeys.1277.184735

**Published:** 2026-04-17

**Authors:** Chengtao Wang, Yuxia Yang, Chen Fang, Xingke Yang, Haoyu Liu

**Affiliations:** 1 Key Laboratory of Zoological Systematics and Application, College of Life Sciences, Hebei University, Baoding 071002, China College of Life Sciences, Hebei University Baoding China; 2 Hebei Basic Science Center for Biotic Interaction, Hebei University, Baoding 071002, China Hebei Basic Science Center for Biotic Interaction, Hebei University Baoding China; 3 Key Laboratory of Zoological Systematics and Evolution, Institute of Zoology, Chinese Academy of Sciences, Beijing, China Institute of Zoology, Chinese Academy of Sciences Beijing China

**Keywords:** Alpha taxonomy, net-winged beetles, Oriental Region

## Abstract

Five new species of *Plateros* Bourgeois, 1879 are discovered from Hainan Island, China, and described as *P. hainanensis***sp. nov**., *P. sinuatus***sp. nov**., *P. elongatus***sp. nov**., *P. dentaticornis***sp. nov**., and *P. spinulosus***sp. nov**. Three previously known species, including *P. belokobylskyi* Kazantsev, 2011, *P. binhanus* (Pic, 1925), and *P. bachmaensis* Kazantsev & Pham, 2026, are recorded to China for the first time. Females of *P. belokobylskyi* and *P. incurvusimimus* Fang, Yang, Yang & Liu, 2024 are identified for the first time. These species are illustrated with their habitus and male genitalia. An identification key to the *Plateros* species found on Hainan Island, China is provided.

## Introduction

*Plateros* Bourgeois, 1879 is one of the largest genera of the beetle family Lycidae, comprising over 900 species worldwide (Kazantsev 2025a), and it is predominantly found in tropical regions (Bocáková 2001). Among these species, nearly one hundred have been documented in the Indochina region (Kazantsev 2021, 2025b; Kazantsev and Pham 2026), with 59 species recorded to China until now (Kazantsev 2025a; Wang et al. 2026). Despite significant contributions from specialists (Bocáková 1997; Kazantsev 1991, 2000, 2011, 2017, 2025a; Fang et al. 2024; Wang et al. 2026), a comprehensive investigation of *Plateros* within the Chinese fauna has yet to be undertaken. Consequently, dozens of unknown species remain to be discovered, particularly in areas where no *Plateros* species have been recorded so far, such as Hainan Island, the southernmost province in China.

During our study, we obtained a large series of *Plateros* material from Hainan Island. Following a thorough examination and comparison with previously documented species from adjacent areas (Fang et al. 2024) and neighbouring countries (Kazantsev 2005, 2020; Kazantsev and Pham 2026), we have identified some common species that are also found in other countries, as well as several new species described herein.

## Materials and methods

The studied specimens are deposited in the Institute of Zoology, Chinese Academy of Sciences, Beijing, China (**IZAS**) and Museum of Hebei University, Baoding, China (**MHBU**).

The specimens were initially softened in water, after which the genitalia of males were dissected. Following dissection, the male genitalia were cleared in a 10% NaOH solution, subsequently examined and photographed in glycerol, and ultimately affixed to a paper card for permanent preservation. Images of the adult specimens were captured with a Canon EOS 80D digital camera, while images of the genitalia were obtained using a Leica M205A stereomicroscope. These images were processed using Helicon Focus v. 7 and edited in Adobe Photoshop CS v. 3.10.0.1.

Measurements were made using Image J v. 1.50i. Body length was measured from the anterior margin of the head to the elytral apex, and width was recorded across the elytral humeri. Pronotal length was determined from the midpoint of its anterior margin to that of its posterior margin, with width measured at its maximum part. The diameter of each eye was assessed at its maximum point, while interocular distance was measured at its narrowest point.

## Results

### Class Insecta Linnaeus, 1758

**Order Coleoptera Linnaeus, 1758**

**Family Lycidae Laporte, 1836**

**Subfamily Lycinae Laporte, 1836**

**Tribe Platerodini Kleine, 1928**

**Genus *Plateros* Bourgeois, 1879**

#### Plateros belokobylskyi

Taxon classificationAnimaliaColeopteraLycidae

Kazantsev, 2011

44E25EFC-0C90-528D-98BA-41D3DE094226

Figs 1A, 1B, 2A–C

Plateros belokobylskyi Kazantsev, 2011: 168, figs 55–56; 2021: 53, figs 47, 155–156.

##### Material examined.

China • Hainan: 1♂ (MHBU), Ledong, Jianfeng, Mingfeng Valley, 3.vi. 2014, leg. J. Y. Su; 1♂ (MHBU), Ledong, Jianfeng, Tianchi, 100 m, 5.vi.2014, leg. J. Y. Su; 1♀ (IZAS), Baisha, Nankai, Nanmaola, 1261 m, 14.v.2009, leg. X. L. Huang.

##### Descriptive notes.

Body length 6.2–8.5 mm (both sexes), width at humeri 1.7–2.8 mm (both sexes).

**Figure 1.**
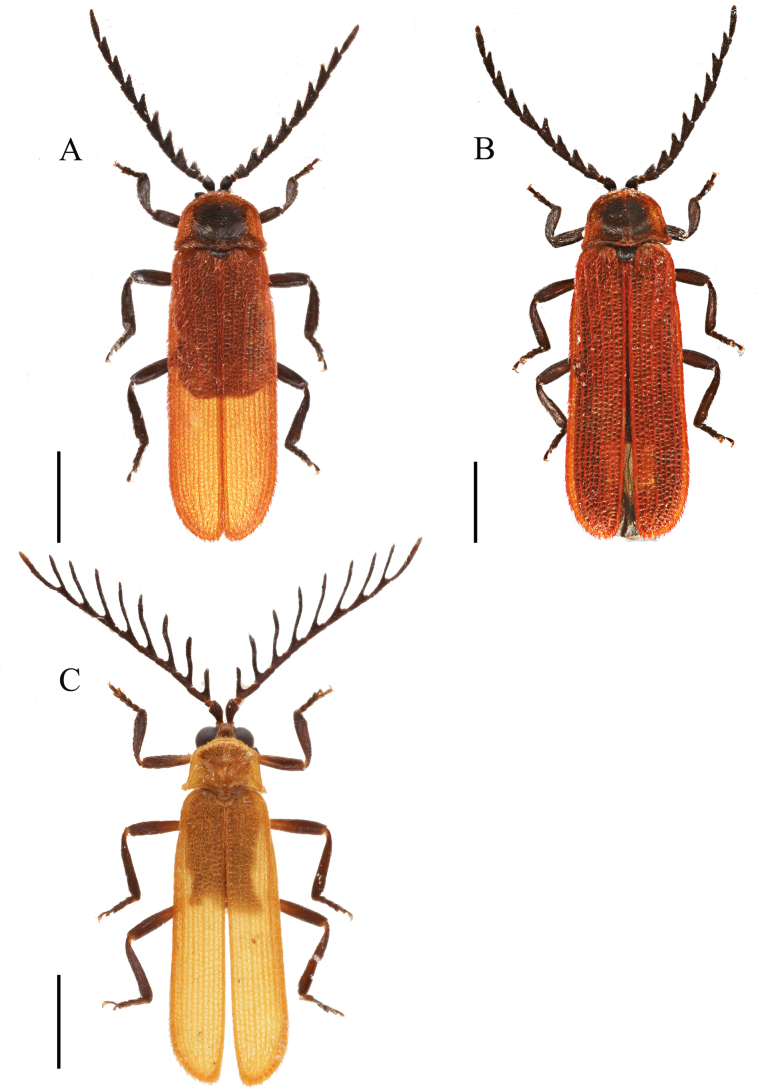
Habitus, dorsal view. **A**. Male *Plateros belokobylskyi* Kazantsev, 2011; **B**. Female *P. belokobylskyi*; **C**. Male *P. binhanus* (Pic, 1925). Scale bars: 2.0 mm.

**Male** (Fig. 1A). Body black. Pronotum red, with a round brown spot on middle disc. Elytra red. Surface covered with short, brownish-yellow pubescence.

***Head*** slightly convex dorsally; eyes small, interocular distance 2.0× eye diameter. Antennae serrate, reaching elytral middle when inclined; antennomeres III–X triangular, with their length 1.0–1.5× width; antennomere III 3.0× longer than II; antennomere IV 1.3× longer than III; antennomeres IV–XI subequal in length.

***Pronotum*** nearly trapezoidal, width 1.6× length; anterior margin slightly arched forward, lateral margins sinuate, posterior margin bisinuate; anterior angles obtusely rounded, posterior angles sharp and postero-laterally projecting.

***Elytra*** widened posteriorly, length 3.0× width, 6.0× longer than pronotum at midlength; primary costae more distinct than secondary costae at humeri.

**Figure 2.**
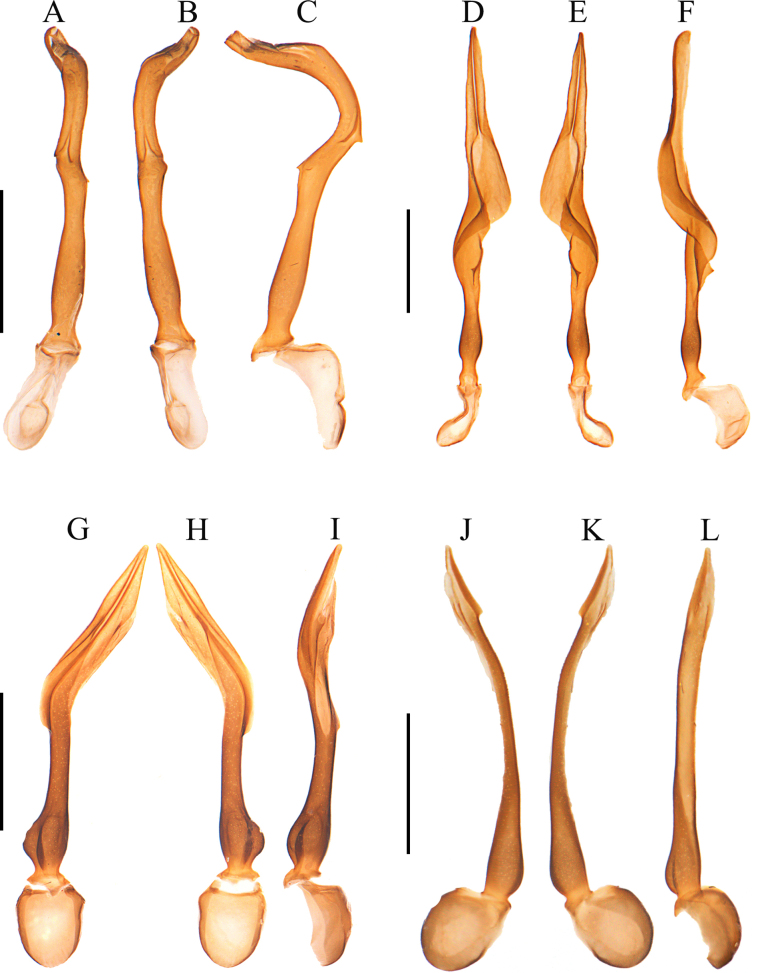
Aedeagus. **A–C**. *Plateros belokobylskyi* Kazantsev, 2011; **D–F**. *P. binhanus* (Pic, 1925); **G–I**. *P. chinensis* Waterhouse, 1879; **J–L**. *P. hainanensis* sp. nov. **A, D, G, J**. Dorsal view; **B, E, H, K**. Ventral view; **C, F, I, L**. Lateral view. Scale bars: 0.5 mm.

***Phallus*** slender, 3.2× as long as phallobase, subequal in width along whole length (Fig. 2A, B), strongly bent ventrally, with apical part at a 90° angle to basal part, truncate at apex, with a small tooth in middle of ventral and dorsal sides, respectively, hardly globally expanded near base (Fig. 2C).

**Female** (Fig. 1B). Similar to male, but eyes smaller; interocular distance 2.2× eye diameter; antennae shorter, reaching basal 2/5 length of elytra when inclined.

##### Distribution.

China (new record: Hainan); Vietnam.

##### Remarks.

The female of this species is reported for the first time. Although the pronotum and aedeagus were illustrated in the original description (Kazantsev 2011), we provide macrophotographs of the habitus and aedeagus herein to exhibit details of these characters.

#### Plateros binhanus

Taxon classificationAnimaliaColeopteraLycidae

(Pic, 1925)

50C696D1-3192-53C9-97F6-2D8FF1C870B2

Figs 1C, 2D–F

Ditoneces binhanus Pic, 1925: 10.
Plateros binhanus : Kazantsev 2021: 55, figs 44, 151–152.

##### Material examined.

China • Hainan: 1♂ (MHBU), Baisha, Nansha, Yashi vill., 333 m, 21.v.2014, leg. J. Y. Su.

##### Descriptive notes.

Body length7.5–7.8 mm (male), width at humeri 1.7–1.9 mm (male).

**Male** (Fig. 1C). Body black. Pronotum and elytra yellow. Surface covered with short, brownish-yellow pubescence.

***Head*** slightly convex dorsally; eyes large, interocular distance 1.2× eye diameter. Antennae pectinate, reaching apical 1/5 length of elytra when inclined; antennomeres III–X with slender lamella, 1.3–2.4× longer than corresponding joint itself; antennomere III 2.3× longer than II; antennomere IV 1.1× longer than III; anntenomeres IV–X subequal in length.

***Pronotum*** trapezoidal, width 1.6× length; anterior margin arched forward, lateral margins sinuate, posterior margin bisinuate; anterior angles obtusely rounded, posterior angles sharp and distinctly projected postero-laterally.

***Elytra*** widened posteriorly, length 2.8× width, 5.4× longer than pronotum at midlength; both primary and secondary costae slightly developed.

***Phallus*** slender, 4.0× as long as phallobase, screw-shaped, tapered apically in apical 1/3, where longitudinally grooved in middle (Fig. 2D, E), widened and twisted in middle part (Fig. 2D–F), with a triangular dorsal protuberance at basal 1/3 (Fig. 2F), feebly globally expanded near base (Fig. 2D–F).

##### Distribution.

China (new record: Hainan); Vietnam, Laos, Thailand.

##### Remarks.

This species was originally described by Pic (1925) and has recently been well illustrated by Kazantsev (2021), enabling its recognition and first recorded occurrence in China.

#### Plateros chinensis

Taxon classificationAnimaliaColeopteraLycidae

Waterhouse, 1879

B0D8D399-F7EB-5602-B5CC-0DE05E639795

Figs 2G–I, 3A, 3B

Plateros chinensis Waterhouse, 1879: 29, pl. 6, fig. 7; Bocáková and Bocak 2007: 219; Fang et al. 2024: 135, figs 1, 2A, B, 3A–C.
Melaneros chinensis : Bocáková 1997: 177, figs 15, 16, 51, 71.
Plateros annamitus Pic, 1921: 7. Synonymised by Kazantsev 2021: 55.
Plateros elisus Pic, 1921: 7. Synonymised by Kazantsev 2021: 55.
Plateros formosanus Pic, 1921: 7. Synonymised by Bocáková 1997: 177.
Plateros formosanus var. nigrolineatus Pic, 1921: 7. Synonymised by Matsuda 2009: 168.
Plateros sycophanta Fairmaire, 1889: 352. Synonymised by Bocáková 1997: 177.
Plateros flavomarginatus Kleine, 1936: 264. Synonymised by Bocáková 1997: 177.

##### Material examined.

China • Hainan: 1♂ (MHBU), Changjiang, Bawangling, 1002 m, 30.v.2014, leg. J. Y. Su; • 1♂ (IZAS), Baisha, Nankai, Shifu vill., 19.00063°N, 109.36757°E, 392 m, 9.v.2009, leg. X. L. Huang; • 1♂ (IZAS), Ledong, Jianfengling, Mingfeng Valley, 18.74393°N, 108.84453°E, 950 m, 3.v.2007, leg. D. Y. Ge; • 1♂ (IZAS), Wenchang, Tongguling, 317 m, 18–19.iii.2008, leg. L. Li; • 1♂ 1♀ (MHBU), Changjiang, Bawangling, 750 m, 5–7.vi.2008, leg. Y. B. Ba & J. T. Lang; • 1♀ (MHBU), Baisha, Nankai, Yashi vill., 21.v.2014, leg. J. Y. Su; • 1♀ (IZAS), Touyuan, Hougang Mangrove Forest, 19.62550°N, 110.79247°E, 1 m, 24.v.2009, leg. X. L. Huang.

##### Distribution.

China (Hainan, Taiwan, Guangxi, Guangdong, Hong Kong, Hubei); Vietnam, Thailand, Cambodia.

##### Remarks.

**Figure 3.**
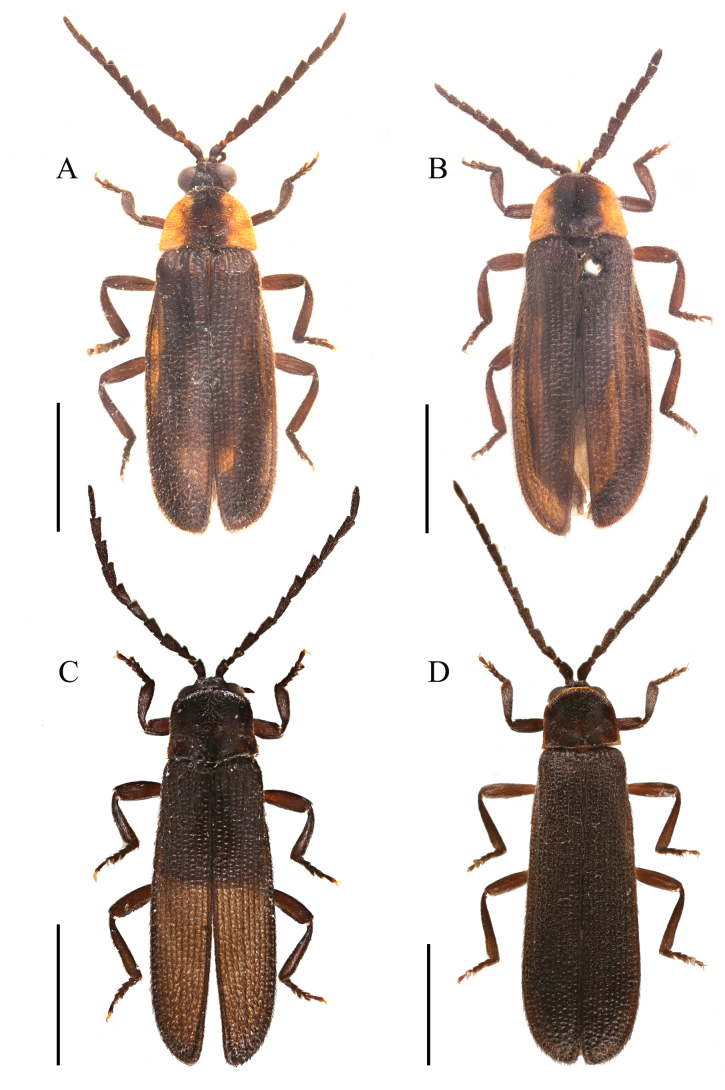
Habitus, dorsal view. **A**. Male *Plateros chinensis* Waterhouse, 1879; **B**. Female *P. chinensis*; **C**. Male *P. hainanensis* sp. nov.; **D**. Female *P. hainanensis* sp. nov. Scale bars: 2.0 mm.

This species is widely distributed in Indochina, including southern China (Fang et al. 2024), but it is found on Hainan Island for the first time in this study. Despite its broad distribution range, the morphology of this species, particularly in terms of general habitus (Fig. 3A, B) and aedeagus (Fig. 2G–I), is quite conservative (Fang et al. 2024: figs 2A, B, 3A–C). This morphological consistency serves as the basis for identifying these specimens from Hainan as *P. chinensis*.

#### Plateros hainanensis

Taxon classificationAnimaliaColeopteraLycidae

Y. Yang, Fang & Liu sp. nov.

E45F6864-1075-528A-BDD3-5BE0EA8EC5CD

https://zoobank.org/DF361B2F-7E68-4B9E-8C66-34054851B59C

Figs 2J–L, 3C, 3D

##### Type material.

***Holotype***. China • Hainan: ♂ (MHBU), Ledong, Jianfeng, Mingfeng Valley, 1–3.vi.2014, leg. J. Y. Su. ***Paratypes***. China • Hainan: 1♂ 3♀♀ (MHBU), Ledong, Jianfeng, Mingfeng Valley, 3.vi.2014, leg. J. Y. Su; • 1♂ 1♀ (MHBU), Ledong, Jianfeng, Mingfeng Valley, 6.vi.2014, leg. J. Y. Su; 3♂♂ 5♀♀ (MHBU), Wuzhishan, Shuiman, 637 m, 13.v.2013, leg. J. Y. Su; • 1♂ 1♀ (MHBU), Ledong, Jianfeng, Botanical Garden, 4.vi.2014, leg. J. Y. Su; • 2♀ (MHBU), Lingshui, Diaoluoshan, 11.v.2014, leg. J. Y. Su; • 1♂ (MHBU), Lingshui, Diaoluoshan, 9.v.2014, leg. L. B. Xiang; • 1♂ (MHBU), Wuzhishan Natural Reserve, 17.v.2014, leg. J. Y. Su; • 1♂ (MHBU), same locality and date as the proceeding, leg. L. B. Xiang; • 1♀ (MHBU), Wuzhishan Natural Reserve, 600–930 m, 16.v.2014, leg. J. Y. Su; • 2♂ (MHBU), Changjiang, Bawangling, 1006 m, 26.v.2014, leg. J. Y. Su; • 1♀ (MHBU), Baisha, Nankai, Yashi Village, 333 m, 20.V.2014, leg. J. Y. Su; • 1♂ (MHBU), Baisha, Yinggezui, 630 m, 22.v.2014, leg. J. Y. Su; • 2♂♂, 1♀ (MHBU), Wuzhishan, Shuiman, 23–25.v.2007, leg. Y.B. Ba & J.T. Lang; • 1♂ (MHBU), Baisha, Nankai, Shibo, 12.vi.2007, leg. Y. B. Ba & J. T. Lang; • 1♂ (MHBU), Baisha, Nankai, Daoda, 24.v.2008, leg. Y. B. Ba & J. T. Lang; • 1♂ 2♀♀ (MHBU), Baisha, Miaocun, 4–5.vi.2007, leg. Y. B. Ba & J. T. Lang; • 1♀ (IZAS), Ledong, Jianfengling, 18.73403°N, 108.87173°E, 1000 m, 4.v.2007, leg. D. Y. Ge; • 1♀ (IZAS), Jianfengling, Tianchi, 18.74482°N, 108.85962°E, 808 m, 22.v.2009, leg. X. L. Huang.

##### Diagnosis.

This species is similar to *P. chinensis*, but it can be easily differentiated from the latter by combination of following characters: pronotum uniformly black (Fig. 3C, D), not with wide yellow lateral margins (Fig. 3A, B); phallus laterally bent, with apical part at a 30° angle to basal part, arrow-like at apex (Fig. 2J, K), not at a 45° angle to basal part and bullet-like at apex (Fig. 2G, H).

##### Description.

Body length 5.2–6.8 mm (both sexes, 5.6 mm in holotype), width at humeri 1.1–1.5 mm (both sexes, 1.3 mm in holotype).

**Male** (Fig. 3C). Body uniformly black. Surface covered with short, brown pubescence.

***Head*** flat dorsally; eyes large, interocular distance 0.8× eye diameter. Antennae serrate, reaching elytral middle when inclined; antennomeres III–X triangular, with their length 1.0–2.0× width; antennomere III 1.5× longer than II; antennomere IV 2.5× longer than III; antennomeres IV–XI subequal in length.

***Pronotum*** nearly trapezoidal, width 1.2× length; anterior margin arched forward, lateral margins slightly sinuate, posterior margin slightly bisinuate; anterior angles obtusely rounded, posterior angle projected posteriorly.

***Elytra*** widened posteriorly, length 2.9× width, 3.8× longer than pronotum at midlength; both primary and secondary costae slightly developed.

***Phallus*** slender, 4.0× as long as phallobase, slightly compressed in middle and laterally bent, with apical part at a 30° angle to basal part, arrow-shaped and sharp at apex (Fig. 2J, K), equal in width and almost straight in lateral view (Fig. 2L).

**Female** (Fig. 3D). Similar to the male, but body stronger, eyes smaller, interocular distance 1.2× eye diameter, and antennae filiform and shorter, reaching basal 1/3 part of elytra when inclined.

##### Etymology.

This species is named after the locality of holotype, Hainan, China.

##### Distribution.

China (Hainan).

#### Plateros incurvusimimus

Taxon classificationAnimaliaColeopteraLycidae

Fang, Y. Yang, X. Yang & Liu, 2024

37CF85E4-1394-591A-BBE2-09857F1E1CF8

Figs 4A, 4B, 5A–C

Plateros incurvusimimus Fang, Y. Yang, X. Yang & Liu, 2024: 145, figs 5J–L, 6C.

##### Type material examined.

***Holotype***. China • Guangxi: ♂ (MHBU), Xing’an County, Maoershan, 4.vi.2011, leg. H. Y. Liu. ***Paratypes***. China • Guizhou: 2♂♂ (MHBU), Maolan County, 23.v.2024, leg. C. Fang & J. L. Miao.

##### Additional material examined.

China • Hainan: 2♂♂ (MHBU), Wuzhishan Natural Reserve, 600–930 m, 16.v.2014, leg. J. Y. Su; • 1♂ 1♀ (MHBU), Wuzhishan Natural Reserve, 781–1563 m, 17.v.2014, leg. J. Y. Su; • 1♀ (MHBU), Wuzhishan, Shuiman, 644 m, 15.v.2014, leg. J. Y. Su; • 1♀ (MHBU), Wuzhishan Shuiman, 637 m, 13.v.2013, leg. J. Y. Su; • 1♂ 1♀ (MHBU), Baisha, Nankai, Yashi vill., 333 m, 21.v.2014, leg. J. Y. Su; • 1♂ (MHBU), Ledong, Jianfeng, Mingfeng Valley, 3.vi.2014, leg. J. Y Su; • 1♂ (MHBU), Lingshui, Diaoluoshan, 300 m, 11.v.2014, leg. J. Y. Su.

##### Descriptive notes.

Body length 4.8–6.0 mm (both sexes), width at humeri 1.5–3.2 mm (both sexes).

**Figure 4.**
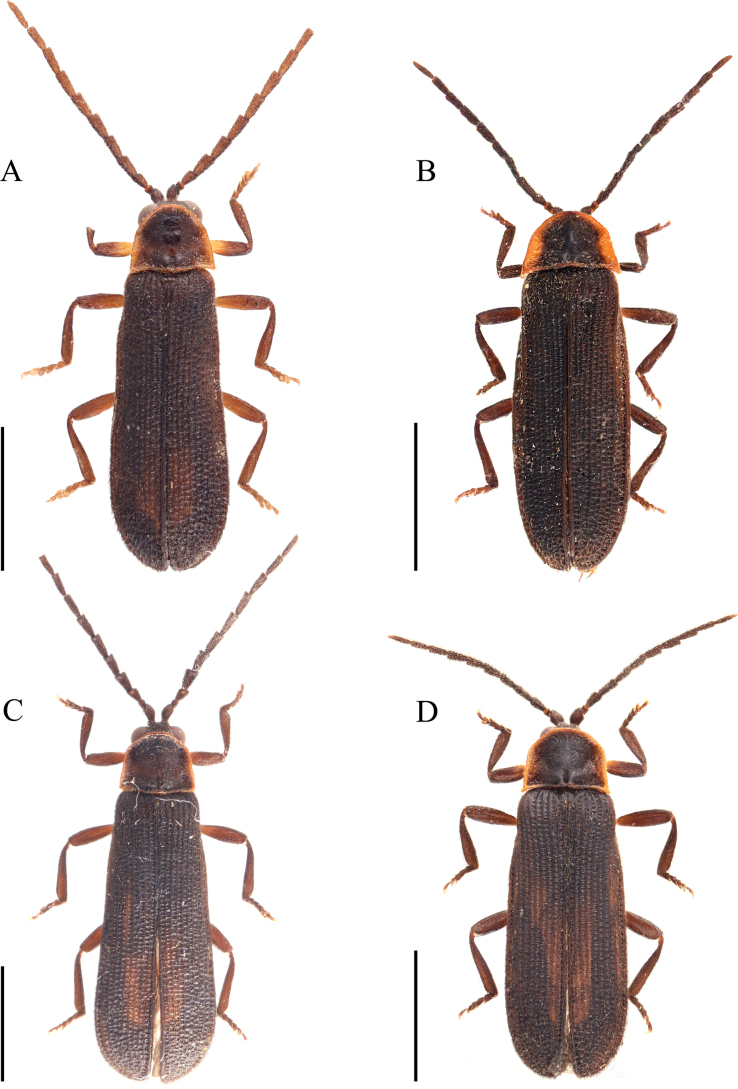
Habitus, dorsal view. **A**. *Plateros incurvusimimus* Fang, Yang, Yang & Liu, 2024; **B**. Female *P. incurvusimimus*; **C**. Male *P. sinuatus* sp. nov.; **D**. Female *P. sinuatus* sp. nov. Scale bars: 2.0 mm.

**Female** (Fig. 4B). Similar to males (Fig. 4A), but eyes smaller, interocular distance 1.2–1.3× eye diameter. Pronotum with wider yellow lateral margins, width 1.8× length. Elytra length 2.5× width, 5.3× longer than pronotum at midlength.

##### Distribution.

China (Guangxi, Guizhou, Hainan).

##### Remarks.

**Figure 5.**
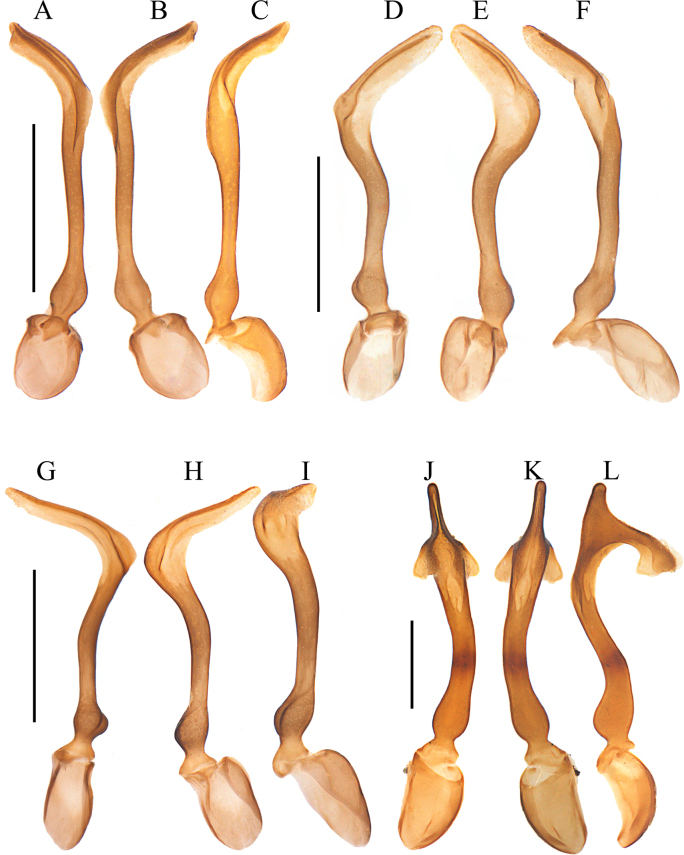
Aedeagus. **A–C**. *Plateros incurvusimimus* Fang, Yang, Yang & Liu, 2024; **D–F**. *P. sinuatus* sp. nov.; **G–I**. *P. elongatus* sp. nov.; **J–L**. *P. bachmaensis* Kazantsev & Pham, 2026. **A, D, G, J**. Dorsal view; **B, E, H, K**. Ventral view; **C, F, I, L**. Lateral view. Scale bars: 0.5 mm.

Based on a thorough examination and comparison with the type material of this species (Fang et al. 2024: fig. 6C), particular regarding the morphology of aedeagus (Fig. 5A–C), the Hainan Island specimens are unequivocally identified as *P. incurvusimimus*. This marks the first recorded occurrence of this species on Hainan Island.

#### Plateros sinuatus

Taxon classificationAnimaliaColeopteraLycidae

Yang, Wang & Liu sp. nov.

30DAA216-8DAD-5274-BFF2-F424B967182F

https://zoobank.org/D0D8A152-51C1-457A-84E5-5915F9CA881E

Figs 4C, 4D, 5D–F

##### Type material.

***Holotype***. China • Hainan: ♂ (MHBU), Wuzhishan, Shuiman, 637 m, 13.v.2013, leg. J. Y. Su. ***Paratypes***. China • Hainan: 2♂♂ 3♀♀ (MHBU), Wuzhishan Natural Reserve, 17.v.2014, leg. J. Y. Su; • 2♂♂ 1♀ (MHBU), Wuzhishan, Shuiman, 637 m, 13.v.2013, leg. J. Y. Su; • 1♀ (MHBU), Lingshui, Diaoluoshan, 950 m, 8.v.2014, leg. J. Y. Su; • 2♀♀ (MHBU), Lingshui, Diaoluoshan, 300 m, 11.v.2014, leg. J .Y. Su; • 1♀ (MHBU), Lengdong, Jianfeng, Mingfeng Valley, 3.vi.2014, leg. J. Y. Su; • 2♀♀ (MHBU), Baisha, Miaocun, 4–5.vi.2007, leg. Y. B. Ba & J. T. Lang.

##### Diagnosis.

This species resembles *P. incurvusimimus*, but it can be easily differentiated from the latter by combination of the following characters: posterior angle of pronotum nearly right-angled (Fig. 4C), instead of acutely angled (Fig. 4A); phallus laterally bent in middle (Fig. 5D, E), instead of bend at apical 1/3 (Fig. 5A, B); apical part of phallus with a ventral notch in lateral view (Fig. 5F), instead of without any notch (Fig. 5C).

##### Description.

Body length 5.1–8.1 mm (both sexes; 5.8 mm in male holotype), width at humeri 1.0–2.8 mm (both sexes, 1.3 mm in holotype).

**Male** (Fig. 4C). Body black; promotum black with red margin; elytra brownish black. Surface covered with short, brown pubescence.

***Head*** flat dorsally; eyes large, interocular distance 1.2× eye diameter. Antennae nearly filiform, reaching elytral half part when inclined; antennomeres III–IV long, triangular, with their length 1.3–1.6× width; antennomeres V–XI parallel-sided; antennomere III 1.9× longer than II; antennomere IV 1.3× longer than III; antennomeres IV and V subequal in length; antennomere VI 1.2× longer than V; antennomeres VI–XI subequal in length.

***Pronotum*** nearly trapezoidal, width 1.2× length; anterior margin arched forward, lateral margins nearly straight, posterior margin slightly bisinuate; anterior angles obtusely rounded, posterior angles nearly right-angled, not posteriorly projecting.

***Elytra*** widened posteriorly, length 2.5× width, 4.6× longer than pronotum at midlength; both primary and secondary costae slightly developed.

***Phallus*** slender, 3.6× as long as phallobase, generally spoon-shaped in ventral and dorsal views, apical half part flattened and rounded at apex, bent laterally at a 45° angle to basal part (Fig. 5D, E), with a pair of lateral ridges along apical 1/3 (Fig. 5D, E) and a notch at subapical 1/4 (Fig. 5F), globally expanded near base (Fig. 5D–F).

**Female** (Fig. 4D). Similar to males, but eyes smaller; interocular distance 1.5× eye diameter. Pronotum with posterior angles posteriorly projecting. Elytra nearly parallel-sided.

##### Etymology.

This species is derived from the Latin *sinuatus* (to wind, bend, curve), referring to its aedeagus shape, which is sinuate in dorsal and ventral views.

##### Distribution.

China (Hainan).

#### Plateros elongatus

Taxon classificationAnimaliaColeopteraLycidae

Y. Yang, Wang & Liu sp. nov.

C6A17204-4D28-5340-B643-895BE744D88F

https://zoobank.org/60DB9666-B6B6-4B5F-AE33-01CD221745BA

Figs 5G–I, 6A

##### Type material.

***Holotype***. China • Hainan: ♂ (MHBU), Wuzhishan Natural Reserve, 781–1563 m, 17.v.2014, leg. J. Y. Su.

##### Diagnosis.

**Figure 6.**
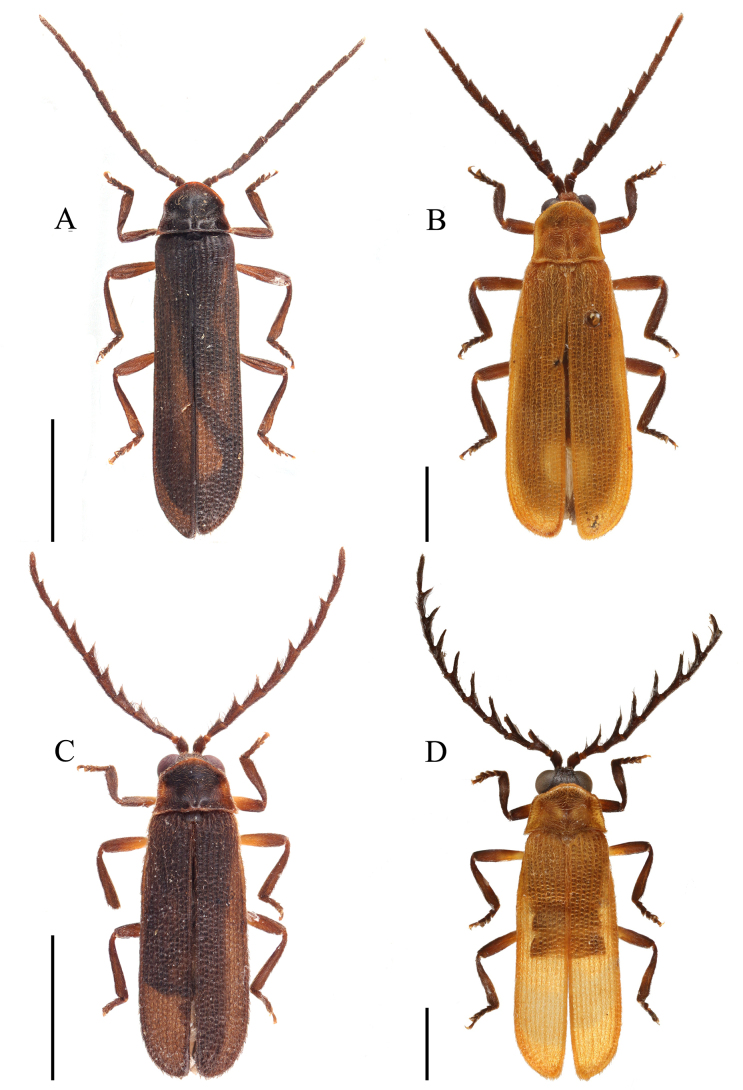
Male habitus, dorsal view. **A**. *Plateros elongatus* sp. nov.; **B**. *P. bachmaensis* Kazantsev & Pham, 2026; **C**. *P. dentaticornis* sp. nov.; **D**. *P. spinulosus* sp. nov. Scale bars: 2.0 mm.

This new species is similar to *P. sinuatus* sp. nov. in the general shape of the aedeagus, but it differs in the following characters: body slender, with elytral length 3.0× width, 5.9× longer than pronotum (Fig. 6A), not stouter, with elytral length 2.5× width, 4.6× longer than pronotum (Fig. 4C); pronotum with lateral margins distinctly emarginate in middle (Fig. 6A), not nearly straight (Fig. 4C); phallus with apical part strongly bent laterally at a 60° angle to basal part (Fig. 5G, H), not moderately bent at a 45° angle (Fig. 5D, E).

##### Description.

Body length 5.8 mm, width at humeri 1.3 mm.

**Male** (Fig. 6A). Body black. Pronotum black, with narrow, brown margins. Elytra brownish black. Surface covered with short, brown pubescence.

***Head*** flat dorsally; eyes large, interocular distance 1.4× eye diameter. Antennae filiform, reaching basal 2/5 part of elytra when inclined; antennomeres III–X cylindrical, with their length 2.7–4.5× width; antennomere III 2.4× longer than II; antennomere IV 1.3× longer than III; antennomere V 1.1× longer than IV; antennomeres V–VIII subequal in length; antennomere VIII 1.1× longer than IX; antennomeres IX–XI subequal in length.

***Pronotum*** nearly trapezoidal, width 1.6× length; anterior margin arched forward, lateral margins emarginate in middle, posterior margin nearly straight; anterior angles obtusely rounded, posterior angles sharp and laterally projecting.

***Elytra*** nearly parallel-sided, length 3.0× width, 5.9× longer than pronotum at midlength; both primary and secondary costae slightly developed.

***Phallus*** slender, 3.5× as long as phallobase, generally hoe-shaped in ventral and dorsal views, apical 2/5 flattened and apically tapered, laterally bent at 60° angle to basal part (Fig. 5G, H), with a longitudinal ridge along apical 1/2, globally expanded near base (Fig. 5G–I).

**Female**. Unknown.

##### Etymology.

This species is derived from the Latin *elongate* (slender), referring to its slender elytra.

##### Distribution.

China (Hainan).

#### Plateros bachmaensis

Taxon classificationAnimaliaColeopteraLycidae

Kazantsev & Pham, 2026

8E4FD992-A6BA-5D48-AEA2-DBFE85007FEE

Figs 5J–L, 6B

Plateros bachmaensis Kazantsev & Pham, 2026: 254, figs 1A, B, 2D, E.

##### Material examined.

China • Hainan: ♂ (MHBU), Changjiang, Bawangling, 750 m, 5.vi2008, leg. Y. B. Ba & J. T. Lang.

##### Descriptive notes.

Body length 8.9–10.2 mm (male), width at humeri 2.3–2.4 mm (male).

**Male** (Fig. 6B). Body black-brown to black. Pronotum, scutellum, and elytra sepia, coxae, and femora brown. Surface covered with short, sepia-coloured pubescence.

***Head*** dorsally flat; eyes large, interocular distance 1.1× eye diameter. Antennae serrate, reaching elytral half part when inclined; antennomeres III–X triangular, with their length 1.5–2.5× width; antennomere III 2.7× longer than II; antennomere IV 1.6× longer than III; antennomeres IV–XI subequal in length.

***Pronotum*** nearly trapezoidal, width 0.9× length; anterior margin arched acutely forward, lateral margins slightly sinuate, posterior margin slightly bisinuate; anterior angles obtusely rounded, posterior angles acute and distinctly postero-laterally projecting.

***Elytra*** widened posteriorly, length 2.9× width, 5.4× longer than pronotum at midlength; both primary and secondary costae slightly developed, subequal in width.

***Phallus*** 3.2× as long as phallobase, abruptly narrowed apically, longitudinally grooved dorsally at apical part, laterally bent at basal 1/3 in dorsal and ventral views (Fig. 5J, K), generally hoe-shaped and sinuate in lateral view, strongly protruding dorsally at subapical part, with protrusion at a 90° angle to trunk, triangular at lateroapical angles (Fig. 5L), appearing like a pair of wings in dorsal and ventral views (Fig. 5J, K), feebly globally expanded near base (Fig. 5J–L).

**Female**. Unknown.

##### Distribution.

China (new record: Hainan); Vietnam.

#### Plateros dentaticornis

Taxon classificationAnimaliaColeopteraLycidae

Y. Yang, Wang & Liu sp. nov.

DE09C696-96A1-56C7-8EBB-680A56A30FB5

https://zoobank.org/E23A34EE-4DD6-4ED2-949B-B922EDCFA030

Figs 6C, 7A–C

##### Type material.

***Holotype***. China • Hainan: 1♂ (MHBU), Baisha, Yinggezui, 630 m, 22.v.2014, leg. J. Y. Su.

##### Diagnosis.

**Figure 7.**
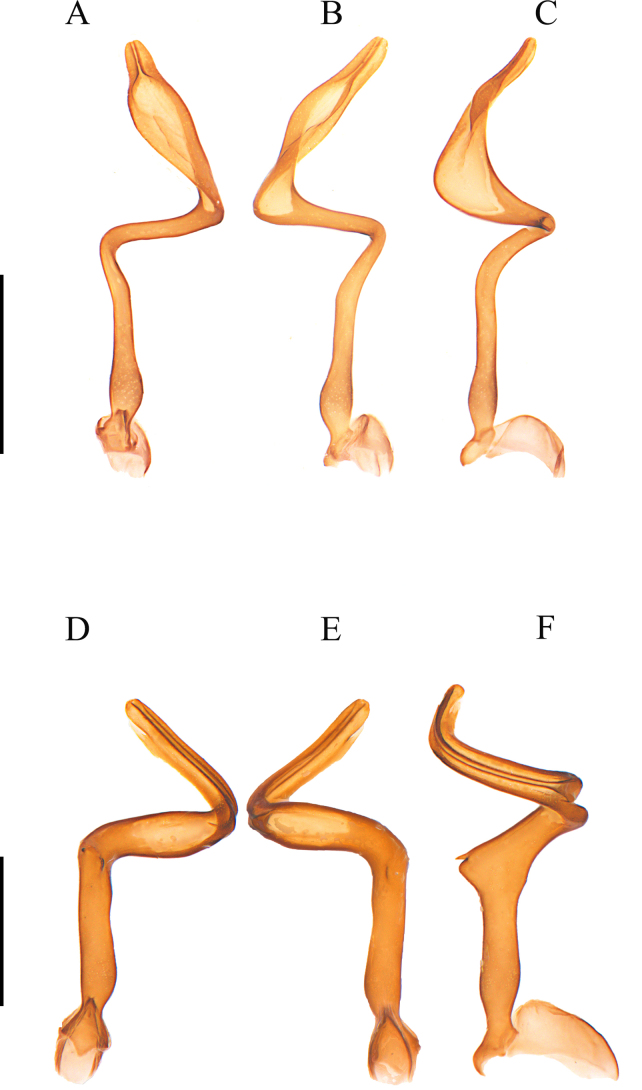
Aedeagus. **A–C**. *Plateros dentaticornis* sp. nov.; **D–F**. *P. spinulosus* sp. nov. **A, D**. Dorsal view; **B, E**. Ventral view, **C, F**. Lateral view. Scale bars: 0.5 mm.

This species is similar to *P. hainanensis* sp. nov. in the appearance, but it can be easily differentiated from the latter by the following: male antennae covered with long pubescence and antennomeres IV–X protruding and very sharp at lateroapical angles (Fig. 6C), versus without any long pubescence and antennomeres IV–X obtuse at lateroapical angles (Fig. 3C); phallus generally spiral (Fig. 7A–C), versus slightly laterally bent (Fig. 2J–L).

##### Description.

Body length 4.5 mm, width at humeri 1.2 mm.

**Male** (Fig. 6C). Body black; pronotum and elytra brown, and basal parts of femora yellow. Surface covered with short, brownish pubescence.

***Head*** flat dorsally; eyes large, interocular distance 1.0× eye diameter. Antennae serrate, covered with long, brownish pubescence, almost reaching elytral apex when inclined; antennomeres III–X long, triangular, length 2.0–2.5× width, with lateroapical angles protruding and sharp at apices; antennomere III 3.5× longer than II; antennomere IV 1.2× longer than III; antennomeres V–XI subequal in length.

***Pronotum*** nearly trapezoidal, width 1.3× length; anterior margin acutely arched forward, lateral margins slightly sinuate, posterior margin slightly bisinuate; anterior angles obtusely rounded, posterior angles posteriorly projecting.

***Elytra*** posteriorly widened, length 3.2× width, 4.9× longer than pronotum at midlength; both primary and secondary costae slightly developed.

***Phallus*** 5.6× as long as phallobase, slender, and generally spiral; apical 1/3 flattened and widened, narrowly rounded at apex, longitudinally grooved dorsally near apex, twisted at a 45° angle to middle part, basal 1/3 nearly straight, strongly bent at a 90° angle to middle part (Fig. 7A, B), feebly globally expanded near base (Fig. 7A–C).

**Female**. Unknown.

##### Etymology.

This species is derived from the Latin *dentatus* (toothed) and *cornus* (horn), referring to its serrate antennae.

##### Distribution.

China (Hainan).

#### Plateros spinulosus

Taxon classificationAnimaliaColeopteraLycidae

Y. Yang, Fang & Liu sp. nov.

C44E01F6-D72E-5139-80F9-3954C4F001EE

https://zoobank.org/C07F4B93-3E22-473A-A10B-118DC92C5EDA

Figs 6D, 7D–F

##### Type material.

***Holotype***. China • Hainan: ♂ (MHBU), Ledong, Jianfeng, Tianchi, 100 m, 5.vi.2014, leg. J. Y. Su.

##### Diagnosis.

This species resembles *P. binhanus* in the appearance, but it can be differentiated from the latter by combination of the following characters: male antennae with long pubescence, versus without long pubescence (Fig. 1C); antennomeres III–X with shorter lamellae (Fig. 6D), versus lamellae longer (Fig. 1C); pronotum with posterior angles feebly posteriorly projected, versus distinctly projecting (Fig. 1C); phallus generally spiral (Fig. 7D–F), versus screw-shaped (Fig. 2D–F).

##### Description.

Body length 8.2 mm (holotype), width at humeri 2.3 mm (holotype).

**Male** (Fig. 6D). Body brownish; pronotum and elytra orange-yellow. Coxae and basal parts of femora yellow. Surface covered with short, brownish pubescence.

***Head*** flat dorsally; eyes small, interocullar distance 1.6× eye diameter. Antennae pectinate, covered with long pubescence, reaching elytral apices when inclined; antennomeres III–X with slender lamellae, 0.7–1.0× longer than corresponding joint itself; antennomere XI filiform; antennomere III 3.1× longer than II; antennomere IV 1.2× longer than III; antennomeres IV–IX and XI subequal in length; antennomere IX 1.3× longer than X.

***Pronotum*** nearly trapezoidal, width 1.6× length; anterior margin acutely arched forward, lateral margins nearly straight, posterior margin slightly bisinuate; anterior angles obtusely rounded, posterior angles acute and feebly laterally projecting.

***Elytra*** widened posteriorly, length 2.9× width, 4.9× longer than pronotum at midlength; both primary and secondary costae slightly developed.

***Phallus*** 5.0× as long as phallobase, generally spiral, apical 1/3 nearly even in width, with a pair of longitudinal dorsal ridges, twisted at a 45° angle to middle part, basal 1/3 nearly straight, strongly bent at a 90° angle to middle part (Fig. 7D, E), with a small spine on portion between middle and basal parts (Fig. 7F), hardly expanded near base (Fig. 7D–F).

**Female**. Unknown.

##### Etymology.

The name of species is derived from the Latin *spinalis* (of or pertaining to a thorn or spine), referring to its phallus with a small spine.

##### Distribution.

China (Hainan).

## Discussion

With the new taxa described here, the number of Chinese *Plateros* species is increased to 67. This species diversity is nearly comparable to that of Vietnam, which has a 73 species documented (Kazantsev and Pham 2026). However, China has significantly greater diversity of *Plateros* species than other neighbouring countries, such as Nepal (36 species; Kazantsev 2025b), Thailand (25 species; Kazantsev 2025c), Laos and Cambodia (20 and six species, respectively; Kazantsev 2021). China encompasses a vast area of 9.6 million km^2^, much larger than Vietnam’s 0.33 million km^2^, which suggests that a greater number of *Plateros* species are likely to be discovered within the Chinese fauna. At present, the documented species within China are predominantly found in the southern regions (Kazantsev 2025a), including Taiwan (19 species), Yunnan (18 species), and Guangdong (six species). Yet, many provinces remain inadequately explored, such as Hainan. In this study, we document the occurrence of *Plateros* species on this island for the first time. While 10 species are reported here, it is anticipated that additional species will be discovered in the future based on some unidentified material in our collection lacking male specimens. It is also expected that some common species from neighbouring countries may be present, such as those newly recorded species found in this study. To assist in the identification of species from Hainan Island, we provide the following key.

### Key to the species of *Plateros* from Hainan, China

**Table:** 

1	Elytra uniformly black (Figs 3A–D, 4A–D, 6A, 6C)	**2**
–	Elytra uniformly red or yellow (Figs 1A–D, 6B, 6D)	**7**
2	Pronotum with wide yellow lateral margins (Fig. 3A, B); apical part of phallus bullet-shaped (Fig. 2G–I)	***P. chinensis* Waterhouse, 1879**
–	Pronotum without or with only narrow yellow margins (Figs 3C, 3D, 4A–D, 6A, 6C); phallus unlike above in shape (Figs 2J–L, 5A–I, 6A–C)	**3**
3	Male antennae serrate; pronotum uniformly black or brown, without yellow margins (Figs 3C, 3D, 6C)	**4**
–	Male antennae filiform; pronotum with narrow yellow margins (Figs 4A–D, 6A	**5**
4	Male antennae covered with long pubescence, antennomeres IV–X with lateroapical angles protruding and very sharp at apices (Fig. 6C); phallus spiral (Fig. 7A–C)	***P. dentaticornis* sp. nov**.
–	Male antennae without any long pubescence, antennomeres IV–X with lateroapical angles never protruding and obtuse at apices (Fig. 3C, D); phallus not spiral, but arrow-shaped (Fig. 2J–L)	***P. hainanensis* sp. nov**.
5	Body slender, elytral length 3.0× width, 5.9× longer than pronotum; lateral margins of pronotum emarginate near middle (Fig. 6A); phallus with apical part strongly bent laterally at a 60° angle to basal part (Fig. 5G, H)	***P. elongatus* sp. nov**.
–	Body stouter, elytral length less than 2.7× width, less than 5.0× longer than pronotum; lateral margins of pronotum not emarginate (Fig. 4A, C); phallus with apical part strongly laterally bent at a 45° angle to basal part (Fig. 5A, B, D, E)	**6**
6	Posterior angles of pronotum acutely projected posteriorly (Fig. 4A); phallus laterally bent at apical 1/3 (Fig. 5A, B), apical part without notch ventrally in lateral view (Fig. 5C)	***P. incurvusimimus* Fang, Y. Yang, X. Yang &Liu, 2024**
–	Posterior angles of pronotum nearly right-angled (Fig. 4C); phallus laterally bent in middle (Fig. 5D, E), apical part with a ventral notch in lateral view (Fig. 5F)	***P. sinuatus* sp. nov**.
7	Pronotum red, with a black marking in middle; elytra red (Fig. 1A, B); phallus with truncate apex (Fig. 2A–C), apical part ventrally bent in lateral view (Fig. 2C)	***P. belokobylskyi* Kazantsev, 2011**
–	Pronotum uniformly yellow, without any black markings; elytra yellow (Fig. 1C, 6B, D); phallus differently shaped (Figs 2D–F, 5G–I, 7D–F)	**8**
8	Male antennae serrate (Fig. 6B); phallus abruptly narrowing apically (Fig. 5J, K), strongly protruding dorsally at subapical part (Fig. 5L)	***P. bachmaensis* Kazantsev & Pham, 2026**
–	Male antennae pectinate (Figs 1C, 6D); phallus differently shaped, never strongly dorsally protruding at subapical part (Figs 1F, 7F)	**9**
9	Male antennae without any long pubescence; antennomeres III–X with longer lamellae than corresponding joint itself (Fig. 1C); phallus screw-shaped (Fig. 2D–F), without any spine at middle (Fig. 2F)	***P. binhanus* (Pic, 1925)**
–	Male antennae with long pubescence; antennomeres III–X with shorter lamellae than corresponding joint itself (Fig. 6D); phallus spiral (Fig. 7D–F)	***P. spinulosus* sp. nov**.

## Supplementary Material

XML Treatment for Plateros belokobylskyi

XML Treatment for Plateros binhanus

XML Treatment for Plateros chinensis

XML Treatment for Plateros hainanensis

XML Treatment for Plateros incurvusimimus

XML Treatment for Plateros sinuatus

XML Treatment for Plateros elongatus

XML Treatment for Plateros bachmaensis

XML Treatment for Plateros dentaticornis

XML Treatment for Plateros spinulosus

## Additional information

### Artificial Intelligence (AI) use

The authors accept full responsibility for the content of the manuscript, including the disclosure of any use of AI.

No AI tools were used in the preparation of this manuscript.

### Author ORCIDs

C. Wang https://orcid.org/0009-0002-8586-6104

Y. Yang https://orcid.org/0000-0002-3118-6659

C. Fang https://orcid.org/0009-0005-0483-8446

X. Yang https://orcid.org/0000-0003-3676-6828

H. Liu https://orcid.org/0000-0003-1383-5560

### Data availability

All of the data that support the findings of this study are available in the main text.
